# Use of a multi-way method to analyze the amino acid composition of a conserved group of orthologous proteins in prokaryotes

**DOI:** 10.1186/1471-2105-7-257

**Published:** 2006-05-18

**Authors:** Alberto Pasamontes, Santiago Garcia-Vallve

**Affiliations:** 1 Chemometrics, Qualimetrics and Nanosensors Group, Analytical and Organic Chemistry Department, Rovira i Virgili University (URV). Campus Sescelades, c/Marcelli Domingo s/n., 43007 Tarragona, Spain; 2 Evolutionary Genomics Group, Biochemistry and Biotechnology Department, Rovira i Virgili University (URV). Campus Sescelades, c/Marcelli Domingo s/n., 43007 Tarragona, Spain

## Abstract

**Background:**

Amino acids in proteins are not used equally. Some of the differences in the amino acid composition of proteins are between species (mainly due to nucleotide composition and lifestyle) and some are between proteins from the same species (related to protein function, expression or subcellular localization, for example). As several factors contribute to the different amino acid usage in proteins, it is difficult both to analyze these differences and to separate the contributions made by each factor.

**Results:**

Using a multi-way method called Tucker3, we have analyzed the amino composition of a set of 64 orthologous groups of proteins present in 62 archaea and bacteria. This dataset corresponds to essential proteins such as ribosomal proteins, tRNA synthetases and translational initiation or elongation factors, which are common to all the species analyzed. The Tucker3 model can be used to study the amino acid variability within and between species by taking into consideration the tridimensionality of the data set. We found that the main factor behind the amino acid composition of proteins is independent of the organism or protein function analyzed. This factor must be related to the biochemical characteristics of each amino acid. The difference between the non-ribosomal proteins and the ribosomal proteins (which are rich in arginine and lysine) is the main factor behind the differences in amino acid composition within species, while G+C content and optimal growth temperature are the main factors behind the differences in amino acid usage between species.

**Conclusion:**

We show that a multi-way method is useful for comparing the amino acid composition of several groups of orthologous proteins from the same group of species. This kind of dataset is extremely useful for detecting differences between and within species.

## Background

Amino acids are not used equally in proteins. In addition to the physical, chemical and biochemical differences between amino acids, which may explain why some amino acids are more used than others, there are also differences in the amino acid composition that are associated with protein function or some characteristic of the species to which they belong. Between proteins from the same species, differences in amino acid composition have been associated with expressivity [1], the chromosomal position of their genes [2,3], hydrophobicity and the number of transmembrane regions [4], subcellular localization [5] and protein function such as differences between ribosomal and non-ribosomal proteins [6]. The availability of complete proteomes from a large number of prokaryotic species has enabled a global comparison of their amino acid compositions to be made and some of the causes of amino acid variability between species to be identified [7-11]. Between species, nucleotide bias and organism lifestyle, especially whether the organisms are mesophilic or thermophilic, are the two main factors behind the variability in amino acid composition [7-11] and even in synonymous codon usage [12]. The methodology used in these analyses involves comparing the means and evaluating which differences are statistically significant or using principal component analysis (PCA) or correspondence analysis to determine which factors influence the amino acid composition of proteins.

Most analyses that compare the amino acid composition between species use whole proteomic averages. However, it could be interesting to compare the amino acid composition of the same group of proteins from different species to check whether the predicted bias is widespread and affects virtually all genes within a genome. Any differences found will then definitely be due to the use of different amino acids between species, not to differences in protein functions. Several authors have used a set of homologous genes from different genomes to show that the observed amino acid bias is a general trend for the evolution of proteins [7,13]. Here we present a similar approach in which we compare the amino acid composition of a set of 64 groups of orthologous proteins common to 62 archaeal and bacterial species. The additional values of our approach, however, are the large number of species used, the fact that the sets of orthologous sequences used correspond to different functional classes, and the procedure used. This procedure consisted of a multi-way method called Tucker3 that analyzes the amino acid variability within and between species using a three-dimensional matrix as an input. In this matrix, the rows represented the frequency with which each amino acid is used, the columns represented groups of orthologous proteins, and the third dimension represented the 62 species analyzed. Briefly, the Tucker3 algorithm decomposes the three-dimensional matrix into a matrix of residuals, three component matrices **A**, **B**, **C **called loadings matrices, and a 3-way core array. This core array defines how the individual loadings vectors interact, providing which factor combinations best represent the data set in terms of explained variability. The order of the core array, i.e. the number of components that are calculated from each loadings matrix, needs to be determined from *a priori *knowledge of the data or by evaluating models with different combinations and choosing the order that provides the most accurate model. See the Methods section for a more detailed explanation of the Tucker3 algorithm.

## Results

To find the optimal order of the Tucker3 model, we studied several combinations of different orders. We found that the 5 × 3 × 3 model was the most suitable because the other models did not improve in fit when higher orders were used. Table 1 shows the elements of the core array obtained in the model of order 5 × 3 × 3 that describe the relationships between the three modes (one for amino acid composition, one for protein function and one for the organisms). The elements are sorted by the percentage of fraction variance that they represent. Only those with the five highest values are shown. With only these five elements, more than 98% of the summed fraction of the variability of the dataset is represented. For all of these five elements, we have also found a biological interpretation and a correlation with some biological character.

### There is a general amino acid usage that is independent of the function or organism analyzed

**Figure 1:**
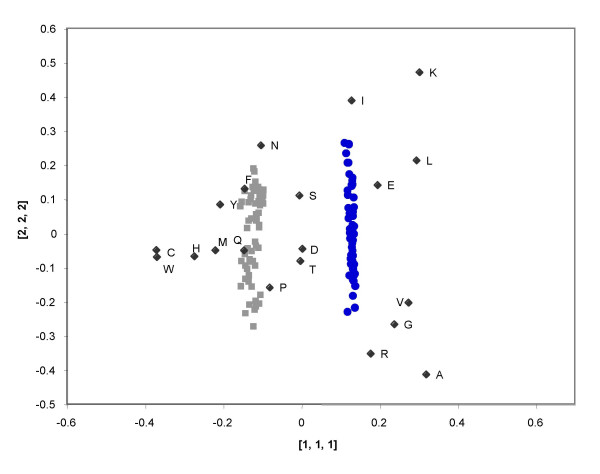
**Triplot of [1, 1, 1] and [2, 2, 2] factors**. Superimposed plot of the [1, 1, 1] and [2, 2, 2] factors showing the amino acid usage variability, independent of the organisms or functions analyzed. This is a combination of three plots: a) a plot of the first and second principal components of the loadings matrix, **A**, related to amino acid variation; b) a plot of the first and second principal components of the loadings matrix, **B**, related to variations associated with functions; and c) a plot of the first and second principal components of the loadings matrix, **C**, related to variations associated with the organism. The blue circles and grey squares represent the organisms and functions, respectively.

The [1,1,1]  factor combination (which means the first component of the amino acid composition, protein function and organism loadings matrices, respectively) explains almost 80% of the variability of the dataset. This value is much higher than the other elements of the core array. Figure 1 shows the representation of the [1,1,1]  against the [2,2,2]  combination (the [2,2,2]  combination is discussed in the next section). Points representing protein function and organism (grey squares and blue circles, respectively) have a similar position in the [1,1,1]  combination and appear as a vertical line. The projections of the amino acid variables onto the horizontal axis show that the amino acids are not used equally. The amino acids on the right (A, V, L, K, G, R, E and I) are the ones used with the highest frequency in the data set. The amino acids on the left (C and W) are the ones used with the lowest frequency. This amino acid composition is very similar to the composition of databases of protein sequences. So, for example, the projections of the twenty amino acids onto the [1,1,1]  element correlates very well (r = 0.917) with the amino acid abundance extracted from Swiss-Prot release 46.5. These results show that there is a general amino acid usage in the proteins analyzed and that this is independent of the protein function or organism to which they belong. This important observation is only possible if a multi-way algorithm is applied to a three-dimensional dataset.

### In the dataset analyzed, ribosomal vs non-ribosomal is the main factor behind variability in the amino acid usage between different protein functions

**Figure 2:**
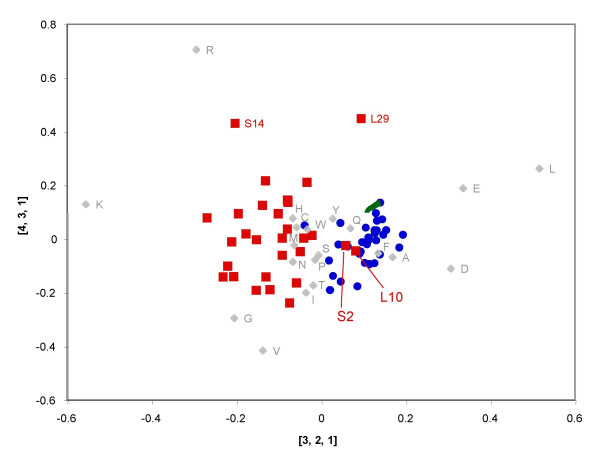
**Triplot of [3, 2, 1] and [4, 3, 1] factors**. Superimposed plot of the [3, 2, 1] and [4, 3, 1] factors showing the amino acid usage variability related to protein function. The red squares and blue circles represent ribosomal and non-ribosomal proteins, respectively. The green triangles represent the loadings matrix of the organisms.

The [3,2,1]  factor combination has a variance fraction of 6.69%. This represents the second element of the core array with the highest variance fraction. This element is represented in figure 2. The loadings values of the organisms, represented by green triangles in figure 2, are localized at the same coordinates. This means that the differences in amino acid composition related to protein function are a general trend and are independent of the organisms analyzed.

Ribosomal and non-ribosomal proteins are represented by red squares and blue circles, respectively. Each group forms a different cluster, which shows that these proteins have different amino acid usages. The good correlation (r = 0.902) between the amino acid differences between ribosomal proteins and non-ribosomal proteins and the projections of the amino acid variables onto the horizontal axis of figure 2 shows that, except for S2, L10 and L29, the ribosomal proteins are distinguishable from the non-ribosomal proteins if we compare their amino acid compositions. Figure 2 shows that the ribosomal proteins use the basic amino acids lysine (K) and arginine (R) and the small hydrophobic amino acids glycine (G) and valine (V) with the greatest frequency and use leucine (L) and the negatively charged amino acids glutamate (E) and aspartate (D) with the lowest frequency. The ribosomal proteins L10, L29, S2 and S14, however, do not cluster with the other ribosomal proteins in figure 2. S2 and L10 appear in the non-ribosomal cluster, and S14 and L29 have a slightly different amino acid composition from those of the other proteins (see figure 2). These differences may be due to the characteristics of these ribosomal proteins in the position or role when the ribosome is formed.

### G+C composition and optimal growth temperature are the main factors behind variability in amino acid usage between organisms

**Figure 3:**
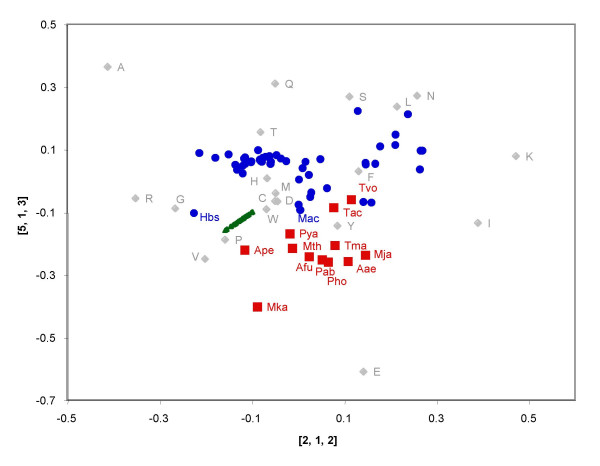
**Triplot of [2, 1, 2] and [5, 1, 3] factors**. Superimposed plot of the [2, 1, 2] and [5, 1, 3] factors showing the amino acid usage variability related to organisms. The red squares and blue circles represent thermophile and non-thermophile organisms, respectively. The green triangles represent the loadings matrix related to protein function. The abbreviations used in this figure are: Hbs, *Halobacterium sp*; Ape, *Aeropyrum pernix*; Mka, *Methanopyrus kandleri*; Mac, *Methanosarcina acetivorans*; Pya, *Pyrobaculum aerophilum*; Mth, *Methanobacterium thermoautotrophicum*; Afu, *Archaeoglobus fulgidus*; Pab, *Pyrococcus abyssi*; Pho, *Pyrococcus horikoshii*; Mja, *Methanococcus jannaschii*; Aae, *Aquifex aeolicus*; Tma, *Thermotoga maritima*; Tac, *Thermoplasma acidophilum *and Tvo, *Thermoplasma volcanicum*.

Figure 3 represents the variability in amino acid usage between organisms. The position of the loadings related to protein function shows that the differences in amino acid composition related to organisms are independent of the proteins analyzed.

The [2,1,2]  combination is the element of the core array with the third highest percentage of variance fraction. The position of the organisms in this element (i.e. the projections of this variable onto the horizontal axis) correlates very well (r = -0.901) with the G+C content of the organisms. This means that the organisms represented on the left of figure 3 (e.g. *Halobacterium sp*, Hbs) have the highest G+C content and those on the right (e.g. *Methanococcus jannaschii*, Mja) have the lowest. Because of the genetic code, the organisms with highest G+C contents use the amino acids glycine (G), alanine (A), arginine (R) and proline (P) [7,8] with the greatest frequency. These amino acids are encoded by codons with a G or C in the first and second codon positions. This is confirmed in figure 3, where the lowest values for the amino acid loadings in the [2,1,2]  factor combination correspond to A, R, G and P. The position of the amino acid valine (V) in this axis, close to the position of proline (P), is also interesting. Valine is encoded by GTN codons, but there are more valines in G+C rich species than expected [8]. This may be due to the many conservative replacements between valine and isoleucine (encoded by AT [A,T,C]) forced by positive GC pressure [8]. On the other hand, figure 3 also shows that lysine (K), isoleucine (I) and asparagine (N) – three amino acids encoded by codons with T or A in the first and second codon positions – are the amino acids that are most used in species with the lowest G+C contents.

The [5,1,3]  combination is also represented in figure 3. The positions of the organisms on the y axis correlate well (r = -0.840) with their optimal growth temperatures. Thermophiles (species with an optimal growth temperature above 60°C) and mesophiles form two clusters in this figure, so they can be distinguished by using their amino acid composition. From the position of the loadings values, we can deduce that the amino acids preferred by thermophiles are mainly glutamate (E) and valine (V). The positions of *Halobacterium sp *(Hbs) and *Methanosarcina acetivorans *(Mac) in figure 3 are interesting. The optimal growth temperatures of these Euryarchaea are below 40°C, so they cannot be classified as thermophiles. However, they have a similar amino acid composition to that of thermophiles and cluster with them in figure 3.

Finally, the [2,2,2]  factor combination shown in figure 1 explains only 0.66% of the variability of the dataset. This factor is a combination of the effects of the G+C content of the organisms and the variability in the G+C contents of the various orthologous genes. Although the G+C content of a gene depends mainly on the G+C content of the organism to which it belongs, there are also variations in the G+C content of genes from the same organism. One of the reasons for these G+C variations within organisms may be compositional amino acid constraints. This is the case of ribosomal proteins, which, because of their compositional amino acid constraints, have a lower G+C content at the second codon position than non-ribosomal proteins. This effect is reflected in the correlation (r = -0.680) between the projections onto the vertical axis of figure 1 of the variables associated with the protein functions and the mean G+C content at the second codon position of each group of orthologous genes.

## Discussion

The high percentage of the variance explained by the [1,1,1]  factor combination shows that the main determinant of the amino acid composition of proteins is independent of the protein function or organism to which they belong. The different uses of amino acids may therefore be due to differences in several biochemical characteristics. However, which amino acid properties influence their usage in proteins is still unknown. Moreover, Jordan et al. [14] have shown that the amino acid composition of proteins is not in equilibrium. By comparing sets of orthologous proteins of closely related genomes from 15 species representing the three domains of life and comparing the fluxes of reciprocal substitutions caused by single-nucleotide replacements, these authors found that cysteine, methionine, histidine, serine and phenylalanine are strong 'gainers' (i.e. their frequency is increasing), and proline, alanine, glutamate and glycine are strong 'losers' (i.e. their frequency is decreasing) [14]. Except for methionine, gainers tend to be under-represented and losers are over-represented [14]. This loser-rich and gainer-poor amino acid composition may be due to the order in which amino acids were recruited into the genetic code [14]. The correlation between the general amino acid frequencies that we observe (the projection of the amino acids onto the x axis in figure 1) and the rate of gain or loss defined by Jordan et al. [14] is only -0.39. The correlation, however, is -0.68 when we compare the general amino acid frequencies and a consensus chronology of incorporation of amino acids into the genetic code defined by Trifonov [15]. This relatively high correlation value means that the order of recruitment of the amino acids into the genetic code can be an additional factor that influences the different use of the amino acids. However, because Trifonov used the amino acid composition of extant proteins as one of the 60 criteria to obtain his consensus chronology of amino acids [15], the above correlation is not unexpected and must be interpreted with caution.

In addition to this general amino acid composition, there are obviously differences in the amino acid composition of proteins due to the function or organism to which they belong. The difference between ribosomal and non-ribosomal proteins is the main factor behind the amino acid usage within species in the data set we analyzed. Shape and charge complementarity rather than sequence-specific interactions are responsible for the specific interactions of most ribosomal proteins with RNA [16]. Because of these interactions, ribosomal proteins prefer positively charged amino acids and avoid negatively charged ones [6]. The mapping of conserved arginines and lysines onto the ribosome structure has revealed that these charged residues frequently form surface patches that reflect RNA-binding sites [6]. The ribosomal proteins L10, L29, S2 and S14, however, do not cluster with the other ribosomal proteins in figure 2. S2 and L10 appear in the non-ribosomal cluster, and S14 and L29 have a slightly different amino acid composition from the other proteins (see figure 2). These differences may be due to the characteristics of these ribosomal proteins in the position or role when the ribosome is formed. Although S2 is one of the largest ribosomal proteins in the 30S subunit, it is very loosely attached to this subunit (only seven out of 236 residues contact with the rRNA) and has the lowest percentage of arginine and lysine in it [16]. It is not unusual, therefore, for S2 to clusters with non-ribosomal proteins. With approximately 60 amino acids, S14 and L29 are the smallest ribosomal proteins in the data set. The short sequence of these proteins influences their amino acid composition and both appear as outliers in figure 2. However, projection of the S14 protein onto the horizontal axis of figure 2 shows that, despite its short length, this protein has some characteristics of the majority of ribosomal proteins. S14 is completely devoid of any globular domain, and most of the protein has an extended coil structure [16]. Although S14 is involved in intimate protein-protein interactions, almost its entire length is involved in RNA contacts and its arginine and lysine contents are similar to those of most ribosomal proteins [16]. S14 is therefore indistinguishable from most ribosomal proteins in the x-axis projection of figure 2. On the other hand, L29 interacts with the L23 protein and with only one of the six domains of 23S rRNA [17]. This characteristic, and its short length, may therefore explain the position of L29 in figure 2.

G+C content and optimal growth temperature are the two factors that most influence differences in amino acid composition between organisms. Analysis of the optimal temperatures of the enzymes extracted from hyperthermophilic organisms showed that thermal resistance was an intrinsic property of these enzymes [18]. Comparative analysis of the amino acid composition of orthologous proteins from several mesophilic and thermophilic organisms indicated some amino acid substitutions that are preferred in thermophiles [18]. However, the small number of sequences analyzed and the fact that factors other than temperature can affect the amino acid composition of proteins revealed the inconsistency of theses results [19]. Comparison of the first completely sequenced genomes of several thermophiles and mesophiles showed that proteins from thermophiles contain higher levels of both charged and hydrophobic residues and lower levels of polar and uncharged ones [20]. Once more complete genomes were sequenced, new analyses were performed using different methods and different datasets [8-10,21-25]. Although these studies show several discrepancies in the role of each amino acid, there is a consensus that glutamate (E) and, to a lesser extent, valine (V) are the amino acids that are more represented in thermophiles than in mesophiles. These were also the amino acids that were most represented in thermophiles when our method was used.

There are greater discrepancies, however, over which amino acids are used with the lowest frequency in thermophiles or with the highest frequency in mesophiles. For example, Singer and Hickey [25] found that these amino acids were A, H, Q and T; Kreil and Ouzounis [8] found that they were Q and T; and Tekaia and coworkers [9] found only Q. These discrepancies indicate that hyperthermophilic and mesophilic enzymes may be very similar – their difference being that hyperthermophilic enzymes are more rigid than mesophilic enzymes [18]. To increase their rigidity, hyperthermophilic enzymes may adopt several strategies but a common rule could be that more charged residues are found in hyperthermophilic proteins, mostly at the expense of uncharged polar residues [18]. Computational, biochemical, and structural evidence now supports the hypothesis that ion pair formation, hydrogen bonds, and hydration, rather than hydrophobic interactions, play important roles in the stabilization of enzymes from extremophiles [26]. Also, we cannot talk of a common amino acid usage in mesophiles because an adaptation to live at intermediate temperatures is unnecessary. When comparing the amino acid compositions of thermophilic and mesophilic proteins, therefore, different datasets and methods obtain different results.

The use of certain amino acids with higher or lower frequencies in thermophiles is important for the thermal stability of their enzymes. However, other factors may contribute to survival at high temperatures. Thermophilic archaea, for example, may be protected by their unique membrane lipids, the use of a reverse gyrase that introduces positive supercoils [27], a DNA repair system [28,29] and the presence of special DNA-binding proteins [29]. One of these thermophilic-specific proteins may be highly basic histone-like proteins that wind and compact DNA into a nucleosome-like structure and thus protect them from heat denaturation [29]. Loss of some of these factors may lead to a lesser ability to grow at high temperatures. This could be the case of the Euryarcheota *Halobacterium sp *(Hbs) and *M. acetivorans *(Mac), two archaea whose optimal growth temperature is below 40°C but that cluster with other thermophilic species in figure 3. The amino acid compositions of these two Euryarchaeota, which are very similar to those of other thermophiles, may be a trace of their past ability to grow at high temperatures. A thermophile-specific NTPase found in 13 thermophilic genomes and absent in 52 mesophilic genomes is present in *M. acetivorans *[30]. This suggests that *M. acetivorans *facultatively could be thermophilic [30]. Although the phylogenetic position of these two archaea and our analysis of the amino acid composition suggest a recent transversion to mesophily in *Halobacterium sp *and *M. acetivorans*, this hypothesis is speculative and needs to be supported by stronger evidence. In this sense, it would be useful to identify proteins present in all thermophilic Euryarchaeota but not in mesophilic Euryarchaeota. One of these proteins is a dsDNA-binding protein called Alba (short for "acetylation lowers binding affinity"), which is present in several thermophilic archaea but not in *Halobacterium sp *or *M. acetivorans *[31]. The correlation of Alba with growth at high temperatures hints at a role for Alba in DNA protection and stability under these conditions [32]. Interestingly, it has been suggested that this protein constrains negative DNA supercoils in a temperature-dependent fashion, which suggests that it may function in chromosomal organization and accessibility [33].

The relationship between genomic G+C content and optimal growth temperature in prokaryotes has been debated recently in the literature [34-37]. Because G:C pairs in DNA are more thermally stable than A:T pairs, it has been suggested that a high G+C content may be a selective response to high temperature. In this sense, a significant correlation has been observed between optimal growth temperature and the G+C content of structural RNAs [35,36]. When open reading frames are analyzed, some studies have concluded that there is no correlation between G+C content and optimal growth temperature [34-36] and others have found a positive correlation among some families of prokaryotes [37]. If this correlation exists, it could be argued that the G+C-content dependence observed in the amino acid composition of prokaryotes is a consequence of their thermophily-dependence. In our dataset, the G+C content at the third codon position and the optimal growth temperature do not correlate significantly (r = 0.081). In addition, the results obtained with the tucker3 algorithm indicate that these two variables are independent. The amino acid variation associated with G+C content and optimal growth temperature corresponds to the second and fifth factor of the amino acid loadings matrix, respectively. Because the principal components obtained with the tucker3 model were constrained to be orthogonal, it can be concluded that the two factors are independent. The correlation observed therefore for the second and fifth factors of the amino acid loadings matrix is only 6.03E-5. Similar arguments can be applied to the second and third factors (those associated with differences in G+C content and optimal growth temperature, respectively) of the organism's loadings matrix. Moreover, the amino acid preferred by thermophiles is glutamate, which is an amino acid encoded by intermediate-GC content codons (GA [A,G]). All this evidence suggests that the amino acid variations related to variations of G+C content and optimal growth temperature are independent and that the observed G+C-dependence is not a consequence of a thermophily dependence.

## Conclusion

We have shown that a multi-way method can be used to analyze differences in the amino acid composition within and between species. This method determines the relative influence of the various factors behind the heterogeneity of amino acid composition in proteins. Also, using a dataset consisting of a group of orthologous proteins present in all the species analyzed ensures that the differences in the amino acid composition between species are related to an intrinsic property of their proteins.

## Methods

### Definition of the data set

Sixty-four orthologous groups of proteins present in 62 archaea and bacteria were imported from the COG database [38]. This data set was chosen because it represents a group of proteins that are present in all the genomes (except for the eukaryotic species and the archaea *Sulfolobus solfataricus*) in the August 2003 version of the COG database [38]. The organisms analyzed [see Additional file 1] comprise various taxonomic groups of bacteria and archaea with different growth temperatures (from mesophiles to hyperthermophiles) and a wide range of G+C contents. The 64 groups of orthologous proteins analyzed [see Additional file 2] have essential functions such as ribosome formation, tRNA synthesis or translation initiation and are therefore conserved in all the organisms we analyzed. Protein sequences of each COG family for the 62 species analyzed were extracted using our own PERL programs. When paralogous sequences were detected, only the largest proteins were retained. In three cases a fusion protein had two of the activities analyzed. These proteins were the HP1198 and jhp1121 from *H. pylori *26695 and *H. pylori *J99, respectively, which contain the two fused beta subunits of a DNA-directed RNA polymerase (COG0085 and COG0086), and the MTH39 protein from *M. thermoautotrophicum*, which contains fused the ribosomal proteins L13 and S9 (COG0102 and COG0103). These fusion proteins were computationally cut into two proteins using information from the comparison of orthologous sequences. So as not to be predicted as horizontally transferred, all the protein sequence in the final dataset was checked using the HGT-Database [39,40]. Using our own PERL programs, we calculated the amino acid composition of each protein of the 64 orthologous groups from the 62 previously defined species. Our data set was therefore a three-dimensional matrix of order (20 × 64 × 62), in which the rows represented the frequency of use of each amino acid, the columns represented the group of orthologous proteins, and the third dimension represented the 62 species analyzed. To analyze the amino acid variability within and between species, the three-dimensional matrix was column mean-centered and analyzed using a multi-way method called Tucker3 [41] developed for the MATLAB (The Mathworks, Natick, MA, USA) environment.

### Analysis of the 3-way data array using the Tucker3 method

In standard multivariate data analysis, data are arranged in a two-way structure (i.e. a table or a matrix) that contains objects and variables. These tables are analyzed with a method such as PCA or correspondence analysis, which enables large amounts of data to be condensed to a few representative variables (called principal components or factors). The projections of objects and variables in the representative principal factors are called scores and loadings matrices, respectively. These matrices are used to identify the similarities and dissimilarities between objects and variables in the data under investigation. In this sense, the scores and loadings plots of different principal components are indispensable for finding patterns and clusters between variables and identifying the variables responsible for the formation of these clusters. They are also indispensable for identifying the variables, or combinations of variables, responsible for the maximum variability between objects. Sometimes, however, the structure of the data set is such that a standard two-way table is not enough to describe it. In these cases, a third mode needs to be added to represent the data set, which can be imagined as a parallelepiped of size r_1 _× r_2 _× r_3_. To apply standard PCA, these three-way data arrays must be unfolded to obtain a two-way data table. This can be done in several ways, depending on what one would like to focus on. Multi-way analysis is the natural extension of multivariate analysis, in which data are arranged in three or more dimensions. There are several multi-way models for analyzing three-way data sets. Here we focus on the Tucker3 model. The origin of this method lies in psychometrics and the pioneering work of Tucker [42]. The algorithm solution for estimating the model was later considerably improved by Kroonenberg and de Leeuw [41].

**Figure 4:**
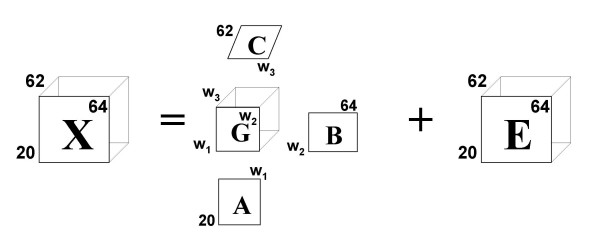
**The 3-way Tucker3 model**. The Tucker3 algorithm decomposes a three-dimensional matrix, **X**, into a matrix of residuals, **E**, three component matrices **A, B, C **called loadings matrices, and a 3-way core array, **G**. The **X **matrix of order (20 × 64 × 62) is the input of the algorithm and contains the frequency with which amino acid is used in each group of orthologous proteins and in each organism. The loadings matrices **A, B **and **C **are similar to the scores or loadings matrices in standard PCA and contain the projections of the **X **matrix in the representative principal factors. The core array, **G**, defines how the individual loadings vectors interact. See the Methods section and reference [43] for more details about the Tucker3 model.

Figure 4 shows the basis of the Tucker3 model, which takes as a starting point a 3-way data array **X** of order (20 × 64 × 62). The tucker3 algorithm decomposes the data array into a matrix **E** of residuals, a 3-way core array **G **of order (w_1 _× w_2 _× w_3_), and three component matrices **A**, **B**, **C **called loadings matrices. In order to use less computation time and make the results easier to interpret, the component matrices of each loadings matrix are usually constrained to be orthogonal. Mathematically, it can be said that

Xijk=∑l=1w1∑m=1w2∑n=1w3ailbjmcknglmn+eijk
 MathType@MTEF@5@5@+=feaafiart1ev1aaatCvAUfKttLearuWrP9MDH5MBPbIqV92AaeXatLxBI9gBaebbnrfifHhDYfgasaacH8akY=wiFfYdH8Gipec8Eeeu0xXdbba9frFj0=OqFfea0dXdd9vqai=hGuQ8kuc9pgc9s8qqaq=dirpe0xb9q8qiLsFr0=vr0=vr0dc8meaabaqaciaacaGaaeqabaqabeGadaaakeaacqWGybawdaWgaaWcbaGaemyAaKMaemOAaOMaem4AaSgabeaakiabg2da9maaqahabaWaaabCaeaadaaeWbqaaiabdggaHnaaBaaaleaacqWGPbqAcqWGSbaBaeqaaOGaemOyai2aaSbaaSqaaiabdQgaQjabd2gaTbqabaGccqWGJbWydaWgaaWcbaGaem4AaSMaemOBa4gabeaakiabdEgaNnaaBaaaleaacqWGSbaBcqWGTbqBcqWGUbGBaeqaaOGaey4kaSIaemyzau2aaSbaaSqaaiabdMgaPjabdQgaQjabdUgaRbqabaaabaGaemOBa4Maeyypa0JaeGymaedabaGaem4DaCNaeG4mamdaniabggHiLdaaleaacqWGTbqBcqGH9aqpcqaIXaqmaeaacqWG3bWDcqaIYaGma0GaeyyeIuoaaSqaaiabdYgaSjabg2da9iabigdaXaqaaiabdEha3jabigdaXaqdcqGHris5aaaa@6430@

where *a*_*il*_, *b*_*jm *_and *c*_*kn *_denote elements of the component matrices **A**, **B **and **C **of orders *20 × w*_1_, *64 × w*_2 _and *62 × w*_3 _respectively, *g*_*lmn *_denotes the elements (*l, m, n*) of the w_1 _× w_2 _× w_3 _core array **G**, and *e*_*ijk *_denotes the error term for element *x*_*ijk *_and is an element of the *20 × 64 × 62 *array **E**. See [43] for more details on Tucker's models.

The number of factors in each of the three modes, i.e. w_1_, w_2 _and w_3_, is determined by the analyst from *a priori *knowledge of the data or by evaluating models with different combinations and choosing the order that provides the most accurate model of **X **[44]. The elements of the core array define how individual loadings vectors in the different modes interact. For example, the [1,1,1]  factor combination means the first component of the amino acid composition, the protein function and the organism modes, respectively. The core array therefore provides a way to interpret the solutions since its squared entries represent the relative importance of each individual factor combination in terms of explained variability. A highly effective way to analyze these combinations of factors is to represent them in a series of bi- or triplots. In our case, triplots consist of the superposition of the representation of two of the principal components for each loadings matrix. These plots can be interpreted as the plot of scores and loadings in standard PCA. By analysing points of the same type (amino acids, functions or organisms), we can analyse the variation observed and the clusters formed. Also, by analysing the mutual positions of points of different types, we can identify which variables are responsible for the variation. Though several successful applications of multi-way models have been demonstrated in quite different areas [43,44], these models have not received much attention in the analysis of proteomic or genomic data. In this paper we show that this kind of algorithm is useful for comparing the amino acid composition of several groups of orthologous proteins.

### Robustness of the model and reliability of the results

Robustness measures the ability of a method to remain unaffected by small variations in variables or procedure. To validate the robustness of our model and check that the results were not a product of chance, we have verified that minor changes in the modelling procedure do not affect our conclusions. Specifically we have verified that the model: (i) was unaffected by small changes in the structural model, and (ii) was unaffected by slight changes such as the elimination at random of some organisms or group of orthologous sequences.

Another important matter is the reliability of the results. Our results have been shown to be reliable by the percentage of summed variance fraction (98.60%) correlated with some biological character, the high value of these correlations, and the consistency of our results with previous studies and our knowledge of the system analyzed.

## Authors' contributions

AP helped to design the study, carried out all the analyses and helped to draft the manuscript. SG-V conceived the study, collected the sequences of the orthologous groups of proteins, supervised the work and wrote the paper. Both authors read and approved the final manuscript.

## Supplementary Material

Additional File 1 Optimal growth temperatures, G+C content and position in the x and y axes of figure 3 of the organisms analyzed. Thermophilic organisms (defined as species with an optimal growth temperature above 60°C) are shown in red. Click here for file

Additional File 2 COG family, function and position on the x and y axis of figure 2 of the genes common to all species analyzed. Click here for file

## Figures and Tables

**Table 1:** Some elements of the core array for a Tucker3 model of order 5 × 3 × 3

**Elements of the core array [aa, funct, org]**	**% of variance fraction**	**% of summed fraction**	**Correlation with biological characters**
[1, 1, 1]	79.98	79.98	amino acid abundance in Swiss-Prot database (r = 0.917)
[3, 2, 1]	6.69	86.67	differences in amino acid composition between ribosomal and non-ribosomal proteins (r = -0.902)
[2, 1, 2]	6.25	92.92	organisms mean G+C content (r = -0.901) and organisms mean G+C at second codon position (r = -0.939)
[4, 3, 1]	3.34	96.26	differences in amino acid composition between ribosomal proteins S14 and L29 and the other proteins (r = 0.892)
[5, 1, 3]	1.68	97.94	optimal growth temperature of organisms (r = -0.840)
[2, 2, 2]	0.66	98.60	mean G+C at second codon position of each orthologous group of genes (r = -0.680)
