# Supplementary material for: Use of a multi-way method to analyze the amino acid composition of a conserved group of orthologous proteins in prokaryotes
Source: BMC Bioinformatics. 2006 May 18;7:257. doi: 10.1186/1471-2105-7-257 (PMC1489954; doi:10.1186/1471-2105-7-257)
Supplement: Additional File 1 — Optimal growth temperatures, G+C content and position in the x and y axes of figure 3 of the organisms analyzed. Thermophilic organisms (defined as species with an optimal growth temperature above 60°C) are shown in red. [file 1471-2105-7-257-S1.PDF]

Supplementary table 1. Optimal growth temperatures. G+C content and position in the x and y axes of figure 3 of the organisms analyzed. Termophilic organisms (defined as species with an optimal growth Temperature higher than 60 ºC) are shown in red.

Organism	Optimal growth T (˚C)	%G+C_T	x axis position	y axis position
Aeropyrum pernix K1	90	57.1	-0.116	-0.220
Agrobacterium tumefaciens	26	59.8	-0.103	0.064
Aquifex aeolicus	95	43.5	0.108	-0.257
Archaeoglobus fulgidus	85	49.0	0.024	-0.241
Bacillus halodurans C-125	30	44.0	0.000	-0.074
Bacillus subtilis	30	43.8	0.025	-0.049
Borrelia burgdorferi	30	28.7	0.262	0.038
Brucella melitensis	37	58.0	-0.117	0.057
Buchnera sp. APS	18	27.3	0.267	0.098
Campylobacter jejuni	37	30.6	0.165	0.057
Caulobacter crescentus	30	67.5	-0.152	0.086
Chlamydia trachomatis	37	41.6	0.015	0.063
Chlamydophila pneumoniae	35	41.1	0.047	0.071
Clostridium acetobutylicum ATCC824	37	31.2	0.140	-0.065
Corynebacterium glutamicum	30	54.5	-0.134	0.037
Deinococcus radiodurans	30	67.4	-0.137	0.053
Escherichia coli K12	37	51.3	-0.080	0.064
Escherichia coli O157	37	51.1	-0.080	0.063
Escherichia coli O157:H7:EDL933	37	51.0	-0.082	0.065
Fusobacterium nucleatum ATCC25586		27.1	0.158	-0.067
Haemophilus influenzae Rd	37	38.4	-0.027	0.065
Halobacterium sp. NRC-1	37	68.5	-0.227	-0.100
Helicobacter pylori 26695	37	39.4	0.144	0.060
Helicobacter pylori J99	37	39.7	0.145	0.055
Lactococcus lactis	30	35.8	0.022	0.021
Listeria innocua	37	37.6	0.027	-0.036
Mesorhizobium loti	26	63.3	-0.116	0.077
Methanobacterium thermoautotrophicum delta-H	65	50.4	-0.013	-0.215
Methanococcus jannaschii	85	31.7	0.145	-0.237
Methanopyrus kandleri AV19	85	60.9	-0.089	-0.402
Methanosarcina acetivorans	37	44.6	0.004	-0.091
Mycobacterium leprae TN	37	60.0	-0.181	0.076
Mycobacterium tuberculosis CDC1551	37	65.5	-0.216	0.091
Mycobacterium tuberculosis H37Rv	37	65.6	-0.215	0.091
Mycoplasma genitalium G37	37	31.4	0.236	0.214
Mycoplasma pneumoniae M129	37	40.3	0.128	0.225
Mycoplasma pulmonis UAB CTIP	37	27.1	0.264	0.098
Neisseria meningitidis MC58	37	52.4	-0.048	0.085
Neisseria meningitidis Z2491	37	52.7	-0.063	0.081
Nostoc sp. PCC 7120	30	42.2	-0.062	0.057
Pasteurella multocida PM70	37	40.8	-0.039	0.073
Pseudomonas aeruginosa PA01	37	67.0	-0.103	0.062
Pyrobaculum aerophilum	100	51.7	-0.018	-0.168
Pyrococcus abyssi	97	44.9	0.052	-0.251
Pyrococcus horikoshii OT3	95	42.0	0.065	-0.259
Ralstonia solanacearum		67.3	-0.119	0.074
Rickettsia conorii Malish 7	37	32.4	0.176	0.112
Rickettsia prowazekii MadridE	37	30.1	0.209	0.116
Salmonella enterica serovar typhimurium LT2	37	52.8	-0.084	0.069

Sinorhizobium meliloti 1021	26	63.3	-0.121	0.050
Staphylococcus aureus N315	37	33.2	0.061	-0.021
Streptococcus pneumoniae TIGR4	37	40.3	0.000	0.007
Streptococcus pyogenes SF320	37	38.9	0.008	0.043
Synechocystis PCC6803	25	48.3	-0.072	0.079
Thermoplasma acidophilum	60	46.9	0.077	-0.084
Thermoplasma volcanium	60	40.9	0.115	-0.059
Thermotoga maritima	80	46.3	0.079	-0.206
Treponema pallidum subsp. pallidum	37	52.9	-0.121	0.026
Ureaplasma urealyticum	37	25.8	0.210	0.150
Vibrio cholerae	37	48.1	-0.061	0.063
Xylella fastidiosa	37	53.4	-0.088	0.101
Yersinia pestis CO92	37	48.3	-0.078	0.075
