# Supplementary material for: Use of a multi-way method to analyze the amino acid composition of a conserved group of orthologous proteins in prokaryotes
Source: BMC Bioinformatics. 2006 May 18;7:257. doi: 10.1186/1471-2105-7-257 (PMC1489954; doi:10.1186/1471-2105-7-257)
Supplement: Additional File 2 — COG family, function and position on the x and y axis of figure 2 of the genes common to all species analyzed. [file 1471-2105-7-257-S2.PDF]

Supplementary table 2. COG family, function and position in the x and y axis of figure 2 of the genes common to all species analyzed.

COG	Function	x axis position	y axis position
COG0008	Glutamyl- and glutaminyl-tRNA synthetases	0.130	0.071
COG0012	Predicted GTPase. probable translation factor	0.102	-0.086
COG0013	Alanyl-tRNA synthetase	0.089	-0.052
COG0016	Phenylalanyl-tRNA synthetase alpha subunit	0.125	0.035
COG0018	Arginyl-tRNA synthetase	0.145	0.018
COG0030	Dimethyladenosine transferase (rRNA methylation)	0.129	0.034
COG0037	Predicted ATPase of the PP-loop superfamily implicated in cell cycle control	0.138	0.138
COG0048	Ribosomal protein S12	-0.271	0.081
COG0049	Ribosomal protein S7	-0.080	0.146
COG0052	Ribosomal protein S2	0.057	-0.023
COG0060	Isoleucyl-tRNA synthetase	0.106	-0.016
COG0072	Phenylalanyl-tRNA synthetase beta subunit	0.137	-0.056
COG0080	Ribosomal protein L11	-0.075	-0.238
COG0081	Ribosomal protein L1	-0.059	-0.162
COG0085	DNA-directed RNA polymerase. beta subunit/140 kD subunit	0.062	-0.025
COG0086	DNA-directed RNA polymerase. beta' subunit/160 kD subunit	0.039	-0.018
COG0087	Ribosomal protein L3	-0.155	-0.190
COG0088	Ribosomal protein L4	-0.042	0.004
COG0089	Ribosomal protein L23	-0.093	-0.059
COG0090	Ribosomal protein L2	-0.212	-0.010
COG0091	Ribosomal protein L22	-0.103	0.096
COG0092	Ribosomal protein S3	-0.081	0.038
COG0093	Ribosomal protein L14	-0.208	-0.138
COG0094	Ribosomal protein L5	-0.023	0.015
COG0096	Ribosomal protein S8	-0.050	-0.045
COG0097	Ribosomal protein L6P/L9E	-0.122	-0.188
COG0098	Ribosomal protein S5	-0.132	-0.140
COG0099	Ribosomal protein S13	-0.133	0.218
COG0100	Ribosomal protein S11	-0.222	-0.100
COG0102	Ribosomal protein L13	-0.093	0.005
COG0103	Ribosomal protein S9	-0.140	0.127
COG0124	Histidyl-tRNA synthetase	0.127	0.033
COG0143	Methionyl-tRNA synthetase	0.129	-0.003
COG0162	Tyrosyl-tRNA synthetase	0.126	0.025
COG0172	Seryl-tRNA synthetase	0.128	0.098
COG0180	Tryptophanyl-tRNA synthetase	0.110	0.008
COG0185	Ribosomal protein S19	-0.179	0.021
COG0195	Transcription elongation factor	0.092	-0.044
COG0197	Ribosomal protein L16/L10E	-0.196	0.096
COG0198	Ribosomal protein L24	-0.233	-0.140
COG0199	Ribosomal protein S14	-0.204	0.433
COG0200	Ribosomal protein L15	-0.153	-0.001
COG0201	Preprotein translocase subunit SecY	0.084	-0.174
COG0231	Translation elongation factor P (EF-P)/translation initiation factor 5A (eIF-5A)	0.019	-0.188
COG0244	Ribosomal protein L10	0.081	-0.042

COG0255	Ribosomal protein L29	0.094	0.450
COG0256	Ribosomal protein L18	-0.079	0.138
COG0258	5'-3' exonuclease (including N-terminal domain of PolI)	0.152	0.036
COG0358	DNA primase (bacterial type)	0.142	0.076
COG0361	Translation initiation factor 1 (IF-1)	-0.041	0.053
COG0441	Threonyl-tRNA synthetase	0.104	0.045
COG0442	Prolyl-tRNA synthetase	0.096	-0.009
COG0480	Translation elongation factors (GTPases)	0.026	-0.135
COG0495	Leucyl-tRNA synthetase	0.089	-0.043
COG0522	Ribosomal protein S4 and related proteins	-0.035	0.212
COG0525	Valyl-tRNA synthetase	0.112	0.001
COG0528	Uridylate kinase	0.044	-0.157
COG0532	Translation initiation factor 2 (IF-2; GTPase)	0.017	-0.078
COG0533	Metal-dependent proteases with possible chaperone activity	0.112	-0.091
COG0541	Signal recognition particle GTPase	0.039	-0.018
COG0550	Topoisomerase IA	0.044	0.062
COG0552	Signal recognition particle GTPase	0.125	-0.088
COG0592	DNA polymerase sliding clamp subunit (PCNA homolog)	0.183	-0.029
COG2890	Methylase of polypeptide chain release factors	0.192	0.019
